# Correction: Registered report: A coding-independent function of gene and pseudogene mRNAs regulates tumour biology

**DOI:** 10.7554/eLife.11802

**Published:** 2015-10-01

**Authors:** Israr Khan, John Kerwin, Kate Owen, Erin Griner, Reproducibility Project: Cancer Biology

Khan I, Kerwin J, Owen K, Griner E, Reproducibility Project: Cancer Biology. 2015. Registered report: A coding-independent function of gene and pseudogene mRNAs regulates tumour biology. *eLife* **4**:e08245. doi: 10.7554/eLife.08245

Published 3 September 2015

Dale Cowley and Kumar Pandya have been removed as the first two authors of the originally published Registered Report and instead their contributions are now listed in the acknowledgments. While they provided feedback and revisions regarding the technical aspects of the experiments to be performed by TransViragen, they do not feel that their contributions are sufficient to warrant authorship. All authors have agreed to this change. In an addition to the acknowledgments, we now state: ‘We thank Dale Cowley and Kumar Pandya, TransViragen Inc, for technical suggestions related to the experiments to be performed’.

