# Correction: Registered report: A coding-independent function of gene and pseudogene mRNAs regulates tumour biology

**DOI:** 10.7554/eLife.13015

**Published:** 2015-11-24

**Authors:** Israr Khan, John Kerwin, Kate Owen, Erin Griner, Reproducibility Project: Cancer Biology

Khan I, Kerwin J, Owen K, Griner E, Reproducibility Project: Cancer Biology. 2015. Registered report: A coding-independent function of gene and pseudogene mRNAs regulates tumour biology. *eLife* **4**:e08245. doi: 10.7554/eLife.08245

Published 3 September 2015

After publication of the Registered report, but before experimental work relating to these changes had commenced, the following revisions have been made.

First, the timing from transfection to generation of lysate for the Western blot procedure described in Protocol 4 has been corrected from 24 hr to 48 hr.

Second, the statistical analysis and power calculations for Protocols 2 and 4 have been expanded to include two additional comparisons, one between siPTEN/PTENP1 and siPTEN, and one between siPTEN/PTENP1 and siPTENP1. This results in an increase in the number of replicates for Protocol 2.

Third, the source of the siRNA used for knockdown of *PTEN* and *PTENP1* in Protocols 2, 3, and 4 has been corrected. The siPTEN Smartpool from Dharmacon, which targets *PTEN* and *PTENP1*, has been included and will be used instead of a mix of siPTEN and siPTENP1.

Finally, the Introduction has also been expanded to include an additional citation (Johnsson et al., 2013).

The article has now been corrected.

